# Maraviroc Reduces Arterial Stiffness in PI-Treated HIV-infected Patients

**DOI:** 10.1038/srep28853

**Published:** 2016-06-29

**Authors:** Stefania Piconi, Daria Pocaterra, Veronica Rainone, Maria Cossu, Michela Masetti, Giuliano Rizzardini, Mario Clerici, Daria Trabattoni

**Affiliations:** 1 Infectious Diseases Unit, L. Sacco Hospital, via G.B. Grassi, 74; 20157 Milano, Italia; 2 Chair of Immunology, Department of Biomedical and Clinical Sciences “L. Sacco”, University of Milan, Milan, Italy; 3 Department of Physiopathology Medical-Surgery and Transplantation, University of Milan, Milan, and Don C. Gnocchi Foundation ONLUS, IRCCS, Milan, Italy

## Abstract

The Δ32-CCR5 deletion of the CCR5 receptor is protective toward coronary artery pathology and myocardial infarction. Maraviroc (MVC), a CCR5 antagonist, was recently introduced in the therapy of HIV infection; we evaluated whether this drug could modulate the atherosclerotic burden in aviremic PI-treated HIV-positive individuals who underwent MVC intensification. Thus, the effect of MVC on intima media thickness, arterial stiffness, metabolic parameters, pro-inflammatory cytokines, endothelial dysfunction, and microbial traslocation markers was analyzed in 6 aviremic PI-treated HIV-positive individuals and were compared to those obtained in 9 additional aviremic PI-treated subjects that were enrolled retrospectively from our outpatients cohort. MVC intensification resulted in a significant reduction in intima media thickness, pulse wave velocity and triglycerides compared to baseline. Notably, MVC was also associated with a significant reduction of IL-6, microbial translocation indexes, sICAM and sVCAM; these changes were maintained throughout the 6 months of MVC intensification. No significant modifications were observed in CD4 counts, HIV viral load, and cholesterolemia. Results herein support a role of CCR5 antagonists in reducing the cardiovascular risk in HIV-infection. The hampering of inflammation, microbial translocation and the improvement of endothelial function could justify the protective role of CCR5 antagonists on atherosclerotic burden.

HIV infection is associated with a high burden of cardiovascular disease that results both from a direct consequence of HIV itself and from the use of highly active antiretroviral treatment and, in particular, of ritonavir-boosted protease inhibitors (PI)^1^. Whereas the aetiology of atherosclerosis in HIV is still unclear, it is well accepted that an excessive production of pro-inflammatory cytokines, likely triggered by microbial translocation, mediates this process^2^. Microbial translocation and high levels of pro-inflammatory cytokines were indeed shown to be closely associated with several cardiovascular risk factors in HIV-infected individuals, including dyslipidemia, insulin resistance, hypertension, coagulation abnormalities, endothelial dysfunction, and carotid atherosclerosis^3^.

Recent results have shown that the Δ32-CCR5 polymorphism, the first described genetic correlate of protection against HIV infection, is associated with an increased plasma concentration of high-density lipoprotein (HDL), a reduction of triglycerides in plasma^4^, and a reduced risk for coronary artery disease and myocardial infarction in the general population^5^.

The likely role played by this genetic polymorphism in atherogenesis was reinforced by data obtained in the animal model. Thus, targeting CCR5 in dyslipidemic mouse models resulted in a significant reduction of both the atherosclerotic burden^6^ and the systemic secretion of proinflammatory Th1 cytokines. Further data indicating that RANTES, the natural CCR5 ligand, is detected on atherosclerotic plaques, myofibroblasts, and endothelial cells and is upregulated in late stages of atherosclerosis in both murine and human plaques lend further support to a role played by CCR5 in atherogenesis^7^. Notably, CCR5 also plays an important critical role in the late phases of atherogenesis, by inducing monocites trapping into the atherosclerotic lesions^8^.

Maraviroc (MVC) is an antiviral drug that acts by targeting the CCR5 receptor, one of the key protein allowing HIV penetration into the target cells. Recent results have shown that the use of this drug reduces the progression of atherosclerosis in a dyslipidemic ritonavir-treated mouse model by interfering with inflammatory cell recruitment into the plaques. Notably, the use of MVC was also shown to result in a decline of plasma lipopolysaccharide in blood^9^,^10^,^11^.

To evaluate whether MVC could have a beneficial effect on the atherosclerotic burden of HIV-infected individuals, we analysed the results of its implementation on intima media thickness, arterial stiffness, metabolic parameters, inflammatory cytokines, endothelial dysfunction and microbial translocation markers in PI-treated HIV-positive individuals.

## Methods

### Study population

Six male HIV-infected individuals (MVC group) were enrolled in this study. Inclusion criteria were: stable PI-treatment for at least one year, undetectable viremia for at least 6 months, R5 HIV viral tropism and Framingham risk score between 10–20%. Exclusion criteria included statin treatment, immunomodulatory therapies in the last 6 months, chronic diseases (e.g. kidney diseases, cancer and immunological diseases).

Marviroc (150 mg × 2 die) was added to current antiretroviral therapy (NRTI+PI) for six months. Patients were evaluated at baseline, 3 and 6 months after MVC intensification. The study was conducted in accordance with “Good Clinical Practice” guidelines and the Declaration of Helsinkin. Individual written consent by all subjects enrolled in the study was collected and ethical approval was granted by the Ethics Committee of L. Sacco Hospital, Milano - Italy.

Nine aviremic patients in stable PI-treatment for at least one year were selected retrospectively from our out-patients cohort as the control group (PI group). These patients had the same inclusion and exclusion criteria of the MVC intensification group and were matched for sex, age and Framingham risk score. Comparisons between the two groups of patients were possible only for CD4+ T lymphocyte counts, metabolic parameters, measurement of carotid IMT and PWV, which were evaluated routinely at three-months intervals in PI-treated individuals. Immunological and microbial translocation parameters were not measured retrospectively in the PI treated group.

### Measurement of carotid IMT and PWV

Measurements of carotid IMT were performed according to the guidelines of the Mannheim IMT Consensus using an ATL HDI-5000 (Philips, Monza, Italy) ultrasound scanner with a 7-MHz linear transducer. Longitudinal electrocardiography (ECG)-triggered images of the right and left common carotid arteries were obtained proximally to the bifurcation and at 1- and 2-cm points, with the patient in the supine position, the head straight, and the neck extended. Measurements were performed on the posterior (far) vessel wall, and images were frozen on the isoelectric line to minimize variability during the cardiac cycle. Pulse wave velocity (PWV) was then obtained using ECG-gated pulse waveforms over the brachial arteries. PWV was calculated as the distance between recording sites measured over the surface of the body, divided by the time interval between the feet of the pressure waves. A single-*blinded* observer performed all the measurements.

### LPS, sCD14, IFABP-1, sVCAM, sICAM, IL-6, and MCP1 plasma concentrations

LPS, sCD14, IFABP-1, sVCAM, sICAM, IL-6, and MCP-1 plasma concentrations were analyzed using commercial ELISA kits (sCD14, sVCAM, sICAM, IL-6, and MCP1: R&D Systems, Minneapolis, Minnesota, USA; Bender MedSystems, Vienna, Austria; LPS: LAL Chromogenic Endopoint Assay, Hycult Biotechnology, Uden, the Netherlands; and IFABP1: Cusabio Biotech, Whuan, China) following the manufacturer’s instructions. Plasma concentration of each protein was calculated in relation to standard curve. These parameters are analysed only in MVC treated group.

### Statistical analyses

Student’s t test was performed to assess differences between means. A *p* value less than 0.05 was considered statistically significant. All statistical analyses were performed with the SAS/STAT, version 9.1 software (SAS Institute, Inc., Cary, North Carolina, USA).

## Results

### Clinical and metabolic data

**Figure 1:**
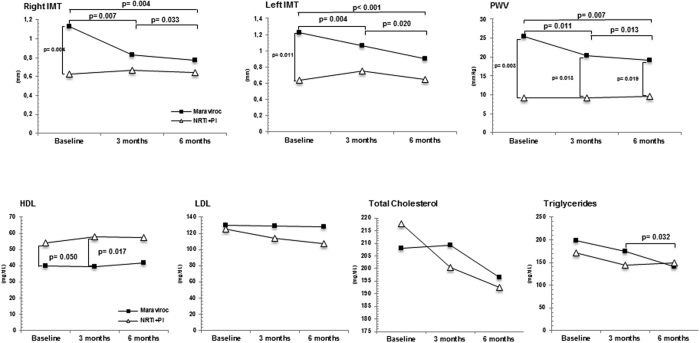
Cardiovascular and metabolic parameters. **Upper panels:** Longitudinal dynamics of Carotid Intima Media Thickness (IMT), Pulse Wave Velocity (PWV) parameters obtained in PI-treated and in MVC-implementation HIV-infected patients. These parameters were analysed at baseline (i.e. after one year of NRTI+PI based therapy), as well as well as 3 and 6 months after baseline (in the Maraviroc implementation group: 3 and 6 months after intensification of therapy with Maraviroc). Mean values and statistical significances are shown. **Lower panels:** Longitudinal dynamic of metabolic parameters (HDL, LDL, Total cholestrol, and Triglycerides) obtained in PI-treated and in MVC-implementation HIV-infected patients. These parameters were analysed at baseline (i.e. after one year of NRTI+PI based therapy), as well as well as 3 and 6 months after baseline (in the Maraviroc implementation group: 3 and 6 months after intensification of therapy with Maraviroc). Mean values and statistical significances are shown.

Clinical and metabolic data of patients enrolled in the study are shown in Fig. 1. No statistical differences were detected between the two groups when CD4+ T lymphocyte counts, HIV viremia, and total and LDL cholesterol were compared throughout the study period. A significant reduction in serum triglycerides (p = 0.032) was observed in the MVC intensification group alone at T6 compared to T3. HDL cholesterol plasma concentration was significantly higher in the PI compared to the MVC intensification group at baseline (p = 0.05) and at T3 (p = 0.017) (Fig. 1) and did not change during the study period in either group.

### Cardiovascular parameters

Cardiovascular parameters were better at baseline in the PI group. Notably, such parameters were not modified during the study period in this group, whereas they were significantly improved during the same time period in those patients whose therapy was intensified with MVC.

Thus, the PI group was characterized at baseline by a significantly better IMT (p = 0.004 right carotid; p = 0.011 left carotid) and a significantly better PWV compared to the MVC intensification group (p = 0.003) (Fig. 1). These parameters nevertheless did not improve during the 6 months study period, (Fig. 1).

**Figure 2:**
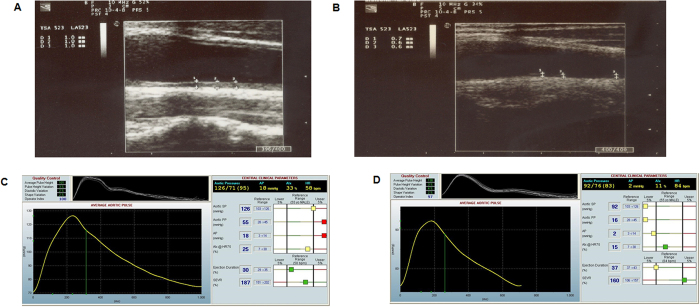
Cardiovascular parameters. **Upper panels:** Carotid Intima Media Thickness (IMT). Representative results obtained in a PI-treated HIV-infected patient before (Panel A) and after (Panel B) six months of therapy intensification with Maraviroc. **Lower panels:** Pulse Wave Velocity (PWV). Representative results obtained in a PI-treated HIV-infected patient before (Panel C) and after (Panel D) six months of therapy intensification with Maraviroc.

MVC intensification, on the other hand, resulted in a significantly reduction of intima media thickness (baseline *vs.* month 3: p = 0.007 right carotid, p = 0.004 left carotid; baseline *vs.* month 6: p = 0.004 right carotid, p < 0.001 left carotid) (Figs 1 and 2A,B). A notable reduction of arterial stiffness, as evaluated by PWV, compared to the baseline values was also evident at month 3 (p = 0.011) and month 6 (p = 0.007) months in the MVC intensification group (Figs 1 and 2C,D). These data indicate that MVC intensification results in a significant reduction of IMT and PWV, suggesting a beneficial effect of this drug on the atherosclerotic burden.

### Microbial translocation

**Figure 3:**
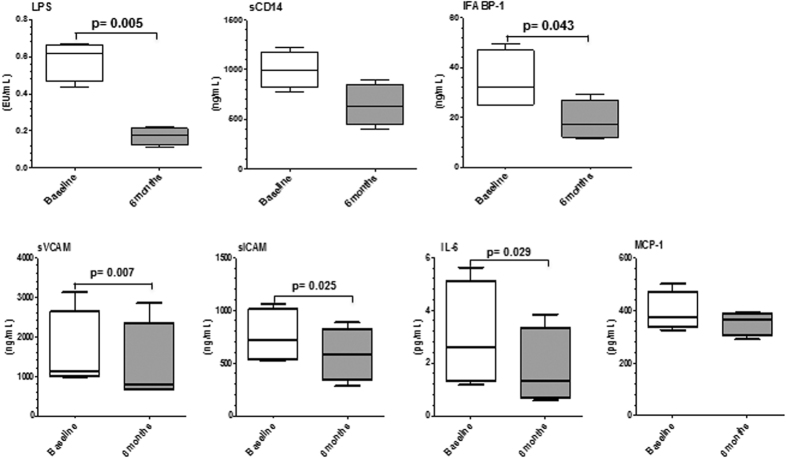
Immunological parameters. LPS, sCD14, IFABP-1, sVCAM, sICAM, IL-6, and MCP-1 plasma concentrations in a group (N = 6) of PI-treated HIV-infected patient before (□) and after () six months of therapy intensification with Maraviroc. The boxes stretch from the 25th to the 75th percentile; the lines across the boxes indicate the median values. Outside values are displayed as separate points. Dots represent all data. Statistical significance is shown.

Plasmatic LPS and iFABP concentrations were significantly reduced by MVC (Fig. 3) (p = 0.005 and p = 0.043, respectively) when the values at month 6 were compared to those obtained at baseline. A similar trend, that nevertheless did not reach statistical significance (p = 0.073) was observed for sCD14 (Fig. 3). These parameters were not evaluated in the PI group.

### Pro-inflammatory cytokines and soluble endothelial adhesion molecules

Plasmatic IL-6 (p = 0.029) and MCP-1 concentrations were reduced after 6 months of MVC treatment (Fig. 3) compared to what was observed at baseline. A similar behaviour was observed in plasmatic sVCAM and sICAM levels (p = 0.007, and p = 0.025, respectively) (Fig. 3). These parameters were not evaluated in PI group.

## Discussion

Results of this study show that six months of MVC intensification in PI-treated individuals results in a positive effect on the atherosclerotic burden, possibly reverting the progression of atherosclerosis. Thus, the use of this antiviral drug was associated with significant improvements in triglycerides profile and cardiovascular parameters (IMT and PWV), as well as in a significant reduction of microbial traslocation, inflammatory markers and membrane adhesion molecules. The observation that no modifications in lipid profile and cardiovascular parameters could be detected in a control group of PI-treated patients who did not undergo MVC intensification strongly suggest that these effects are due to the use of MVC.

Results obtained in the Merit study^12^ showed that MVC intensification results in an improvement of lipid parameters in PI-treated patients, suggesting a protective effect of this drug on dismetabolic damages induced by protease inhibitors. Our data confirm those stemming from the Merit study by indicating that the metabolic pro-atherogenic observed in our patients could be reversed by MVC. Notably, data herein shed further light on the beneficial effects of MVC on cardiovascular risk by showing that the addiction of MVC to a PI-based regimen results in a significant reduction of the intima media thickness and improvement in PWV; these parameters did not change in the PI group during the observation period. These data for the first time confirm in the clinical setting the results obtained *in vitro* and in animal models^5^ according to which MVC is able to limit PI treatment induced atherosclerosis progression, and support cohort study data reporting a lower incidence of cardiovascular events in CCR5-Δ32-carrying patients^4^.

The significant reduction seen in the plasma levels of membrane adhesion molecules (expression of endothelial dysfunctions) further confirms that MVC intensification in PI-treated patients is associated with an improvement of endothelial functions and highlight the protective effect of CCR5 inhibitors on antiretroviral induced vascular damages. Notably, MVC resulted in a significant reduction of microbial translocation as well. This observation is important considering that such translocation is believed to trigger the immunoactivation that characterizes HIV infection, and is considered to be the major culprit of atherogenesis^13^. Thus, MVC was associated with a significant reduction of LPS, iFABP and, possibly as consequence, of IL-6, MCP-1 and sCD14. All these factors play a crucial role in atherogenesis^13^, and their reduction during MVC intensification underlines the positive effects of CCR5 inhibitors in reducing the progression of the atherosclerosis process in HIV-infected patients.

Recent results indicate that an increase of CCR5+/CD8+ T lymphocytes heralds ischemic cardiovascular events in HIV-infected patients on suppressive cART. These CCR5-expressing cells are trapped in the atherosclerotic plaque where they have a pathologic effect, the use of MVC would impede this process and could, therefore, also be envisioned as a possibly preventative agent against the development of acute coronary syndrome (ACS)^14^.

The small number of examined individuals and the observation that the control group was retrospectively enrolled could apparently hamper the validity of these results. It is nevertheless important to observe that a statistical improvement in cardiovascular parameters was only observed in the MVC groups, thus supporting the beneficial effect of MVC on such parameters.

In conclusion, data herein, although preliminary, suggest a protective effect of MVC on inflammatory, metabolic and endothelial parameters associated to the atherogenesis process in HIV-infected individuals. Further analyses on the relationship between MVC and endothelial functions could lead to the identification of new and interesting fields of his therapeutic application.

## Additional Information

**How to cite this article**: Piconi, S. *et al*. Maraviroc Reduces Arterial Stiffness in PI-Treated HIV-infected Patients. *Sci. Rep.* **6**, 28853; doi: 10.1038/srep28853 (2016).
